# The Cardioprotective Effects of Nutritional Ketosis: Mechanisms and Clinical Implications

**DOI:** 10.3390/nu16234204

**Published:** 2024-12-05

**Authors:** Claudia Venturini, Lucia Mancinelli, Giulia Matacchione, Fabiola Olivieri, Roberto Antonicelli

**Affiliations:** 1 Clinical Nutrition Unit, IRCCS INRCA, 60127 Ancona, Italy; 2 Cardiology Unit, IRCCS INRCA, 60127 Ancona, Italy; 3 Clinic of Laboratory and Precision Medicine, IRCCS INRCA, 60127 Ancona, Italy; 4 Department of Clinical and Molecular Sciences (DISCLIMO), Università Politecnica delle Marche, 60126 Ancona, Italy

**Keywords:** ketogenic diet, inflammation, beta-hydroxybutyrate, cardiovascular risk, obesity

## Abstract

Cardiovascular diseases (CVDs) persist as the primary cause of death worldwide, accounting for roughly 17.9 million fatalities each year. The prevalence of obesity, metabolic syndrome, and type 2 diabetes (key risk factors for CVD) continues to escalate at an alarming rate, necessitating novel therapeutic strategies to address this global health crisis. Nutritional ketosis, induced through ketogenic diets, modified fasting, intermittent fasting, and medium-chain triglyceride (MCT) oil consumption, has garnered attention for its potential cardioprotective effects. Ketosis is a metabolic state in which the body, due to a significantly reduced intake of carbohydrates, shifts its primary energy source from glucose to ketone bodies, i.e., beta-hydroxybutyrate (BHB), acetoacetate, and acetone, which are produced in the liver from fatty acids. This review examines the mechanisms by which ketone bodies, particularly BHB, mitigate cardiovascular risk. We focus mainly on the anti-inflammatory and antioxidative properties of BHB and summarize recent evidence to highlight the clinical relevance of ketosis in cardiometabolic health.

## 1. Introduction

Cardiovascular diseases (CVDs) remain the primary cause of mortality globally, responsible for approximately 17.9 million deaths annually, as reported by the World Health Organization (WHO, 2023). Obesity is a key risk factor for CVD and continues to escalate at an alarming rate, necessitating novel therapeutic strategies to address this global health crisis (WHO, 2023). Indeed, obesity elevates total blood volume and cardiac output, thereby increasing cardiac workload. Patients with obesity typically exhibit higher cardiac output with reduced total peripheral resistance, leading to increased filling pressures, chamber dilation, concentric remodeling, and left ventricular hypertrophy (LVH). These abnormalities enhance the risk of ischemic heart disease, heart failure, atrial fibrillation, complex ventricular arrhythmias, and both diastolic and systolic dysfunction. Moreover, visceral obesity directly increases cardiovascular risk factors such as dyslipidemia, type 2 diabetes, arterial hypertension, and sleep disorders [1]. One of the main causes of CVD is visceral adiposity, which promotes systemic and vascular inflammation implicated, for example, in all stages of atherosclerosis, from initial fatty streak formation to atherothrombosis [2,3].

Particularly, a major subset of adults over the age of 65 is now classified as having sarcopenic obesity, a high-risk geriatric syndrome predominantly observed in an aging population that is at risk of synergistic complications from both sarcopenia and obesity [4]. Sarcopenic obesity involves an age-associated increase in adiposity and reduction in muscle mass and function, posing a major health risk to older adults and presenting management challenges in clinical settings.

Recently, the ketogenic diet has gained attention for its multifaceted effects in the prevention and treatment of CVD. The ketogenic diet (KD) primarily consists of high fat intake, moderate protein consumption, and low carbohydrate intake [5].

The ketogenic diet is extensively studied for its neuroprotective properties, particularly in epilepsy, Alzheimer’s, and Parkinson’s disease [6,7,8].

Moreover, it has been proposed as an adjunctive therapy for cancer treatment. By depriving cancer cells of glucose, their primary energy source, the KD may inhibit tumor growth [9].

Notably, the KD has been shown to improve the blood lipid profile, often outperforming other diets, and displays significant anti-inflammatory and thus cardioprotective properties. The state of ketosis reduces inflammation through the elimination of simple sugars, carbohydrate restriction, and omega-3 fatty acid supplementation. Ketone bodies (i.e., beta-hydroxybutyrate (BHB), acetoacetate, and acetone) also serve as “rescue fuel” for the compromised heart, positively influencing its metabolism. Moreover, the ketogenic diet enhances endothelial function and helps prevent vascular aging, and it has favorable effects on blood pressure and other CVD risk factors, partly due to weight reduction [10].

Given the compelling evidence supporting the cardioprotective effects of nutritional ketosis, this review aims to delve deeper into the underlying mechanisms by which ketone bodies, particularly BHB, confer cardiovascular benefits. We will also explore the various methods to achieve nutritional ketosis and discuss the clinical implications of these findings.

## 2. Visceral Adipose Tissue: A Source of Inflammation

Although the link between CVD and obesity has consistently and strongly been proven, the one-size-fits-all approach should not be used with obesity, because there are relevant factors largely affecting the CVD prognosis of individuals with obesity [11]. The quality and functionality of adipose tissue (AT) significantly influence cardiometabolic risk, with the negative impacts of dysfunctional AT expansion in obesity occurring at both local and systemic levels. 

**Figure 1:**
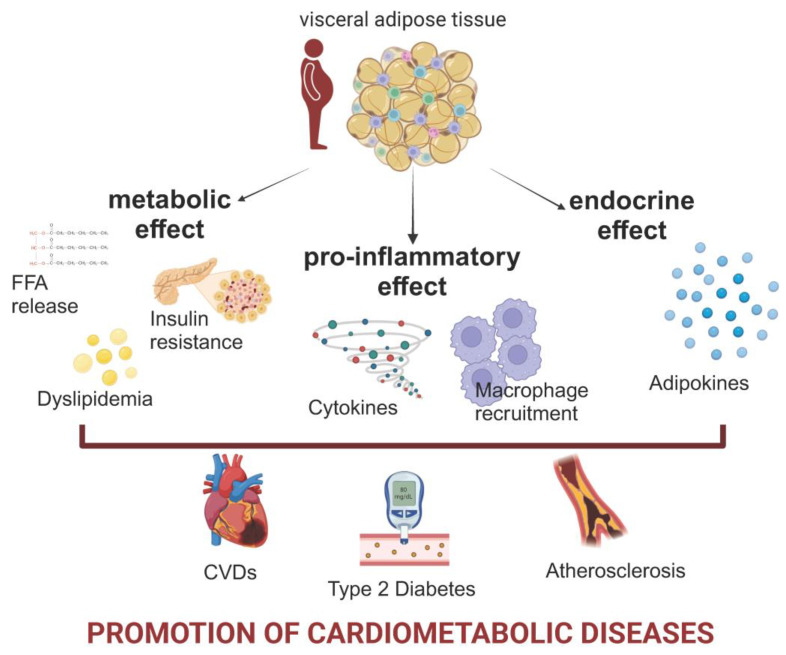
The central role of visceral adipose tissue (VAT) in obesity-related cardiometabolic risks. VAT dysfunction contributes to insulin resistance, increased lipolysis, and free fatty acid (FFA) release, disrupting lipid and glucose metabolism. As an endocrine organ, VAT secretes adipokines such as leptin, adiponectin, and resistin, but in obesity, this balance shifts, with decreased beneficial adipokines and increased harmful ones. VAT also drives systemic inflammation by releasing pro-inflammatory cytokines like TNF-α and IL-6 and recruiting macrophages. These processes link VAT dysfunction to the development of type 2 diabetes, atherosclerosis, and coronary artery disease (CAD). Arrows indicate the pathways connecting VAT to these systemic effects.

Indeed, visceral AT (VAT) contributes to metabolic and cardiovascular diseases by promoting metabolic dysfunction, insulin resistance, and systemic inflammation (Figure 1) [12]. VAT is made up of large, insulin-resistant adipocytes that lead to excessive lipolysis, releasing free fatty acids (FFAs) into the liver, disrupting lipid and glucose metabolism, and contributing to hyperinsulinemia and type 2 diabetes [13]. VAT also contains a higher density of hormone receptors like glucocorticoid and androgen receptors, which cause VAT to increase with age, especially in men [14]. Additionally, VAT acts as an endocrine organ, secreting adipokines such as leptin, adiponectin, omentin, resistin, and visfatin, which regulate metabolism [15,16]. In obesity, these adipokines become dysregulated, with the quantities of beneficial ones like adiponectin being decreased and those of harmful ones like resistin being increased, further worsening insulin resistance and metabolic disorders.

Moreover, the expansion of VAT is closely linked to inflammation. VAT attracts macrophages and immune cells, which release pro-inflammatory cytokines like TNF-α and IL-6, contributing to both local and systemic low-grade inflammation [14]. These pro-inflammatory factors propagate to the liver, exacerbating metabolic disturbances such as steatosis and insulin resistance [17].

We previously demonstrated that specific polymorphisms related to pro-inflammatory cytokines, i.e., IL-6 and TNF-alpha, in patients with acute coronary syndrome (ACS) were associated with an increased mortality rate at a one-year follow-up [18]. Notably, elevated IL-6 levels were linked to the severity of CAD, and increased levels of C-reactive protein (CRP) were associated with an increased one-year risk of death in male patients with ACS, supporting the hypothesis that inflammation is a critical driver in the pathogenesis and progression of atherosclerosis [19].

We recently demonstrated that in morbid obesity, the expansion of visceral adipose depots involves an increased burden of macrophages with a senescent-like phenotype that may promote a pro-inflammatory profile and impair insulin signaling in adipocytes, supporting a framework in which senescent macrophages fuel obesity-induced systemic inflammation and possibly contribute to the development of insulin resistance [20].

Recently, a novel study emphasized that systemic inflammation plays a crucial role in the progression of coronary artery disease (CAD) and its severity. The analysis showed that patients with higher levels of systemic inflammation had a higher prevalence of coronary heart disease (CHD), and these findings were consistent across various subgroups, including age, gender, and other demographic factors [21].

We also analyzed monocyte polarization, which is a process closely associated with the initiation of inflammatory responses. Our analysis revealed a significantly elevated proportion of intermediate and non-classical CD80+ monocytes, indicative of a shift toward a pro-inflammatory phenotype in patients experiencing acute myocardial infarction (AMI) compared to in older control subjects [22].

Overall, the relationship between inflammation and cardiovascular outcomes highlights the importance of addressing inflammatory factors to prevent or mitigate CVD risks.

## 3. Strategies for Obesity Prevention and Weight Control

### 3.1. Mediterranean Diet

There is evidence that even modest weight loss, such as 5–10% of initial weight, can reduce cardiovascular disease risk even when the patient remains in the obese range [23]. Look AHEAD is the first study to demonstrate the benefits of weight loss derived from an intensive lifestyle intervention through 4 years of follow-up in a large cohort of individuals who were overweight and obese with type 2 diabetes [24].

Several studies have demonstrated that weight loss and adherence to a healthy diet are critical in achieving the cardioprotective effects of a diet. A meta-analysis emphasized the impact of the Mediterranean diet on cardiovascular risk factors such as cholesterol levels, blood pressure, and glycemic control, showing risk reduction particularly for patients with type 2 diabetes [25]. Similarly, a systematic review and meta-analysis confirmed that a Mediterranean diet, especially when combined with efforts to reduce weight, can reduce cardiovascular events and mortality. The Mediterranean diet’s benefits were particularly prominent in populations with dietary adherence that also focused on reducing body mass index and managing overall weight, showcasing how weight loss enhances the cardioprotective effects of this diet [26]. Additionally, another review pointed out that weight loss through dietary changes was linked to a reduction in CVD risk factors, emphasizing that both weight management and a Mediterranean dietary pattern are pivotal for optimal heart health outcomes [27,28,29].

Furthermore, a study linked the Mediterranean diet to a lower incidence of cardiovascular diseases over a 12-year period in the EPIC-NL cohort, reinforcing the evidence of long-term cardiovascular protection associated with this dietary pattern [30]. To summarize, these studies indicate that weight loss offers robust cardioprotective benefits, making diet adherence an effective approach for both the primary and secondary prevention of cardiovascular diseases.

### 3.2. Ketogenic Diet

Traditional dietary interventions have focused on reducing fat intake, but emerging research suggests that nutritional ketosis, a metabolic state achieved through ketogenic diets, intermittent fasting, and other methods offer profound benefits for cardiovascular health.

Nutritional ketosis is characterized by elevated levels of ketone bodies, such as beta-hydroxybutyrate (BHB), which serve as alternative energy sources when carbohydrate intake is significantly reduced. The review by Matsuura et al. highlights that ketone bodies, particularly BHB, serve as efficient substrates for ATP production in the heart, especially under conditions of heart failure (HF). During HF, the heart’s reliance on fatty acids and glucose for energy is impaired, and ketone bodies can provide an alternative, efficient fuel source. Enhanced cardiac ketone utilization is facilitated by the upregulation of key ketolytic enzymes, such as BDH1 and SCOT, which support increased oxidation of BHB and AcAc, thereby maintaining ATP production and improving cardiac energetics [31].

Recent studies have highlighted the multifaceted roles of BHB, not only as a fuel source but also as a “signaling molecule” with significant therapeutic potential. BHB has been shown to exert anti-inflammatory effects, enhance endothelial function, provide antioxidative benefits, and modulate lipid metabolism—mechanisms that collectively contribute to reduced cardiovascular risk.

The resurgence of interest in ketogenic diets and other ketosis-inducing strategies is supported by a growing body of clinical evidence demonstrating their efficacy in improving metabolic health markers. For instance, ketogenic diets have been associated with significant reductions in body weight, improvements in glycemic control, and favorable shifts in lipid profiles, all of which are crucial for cardiovascular health [32,33]. Moreover, intermittent fasting and modified fasting regimens have been shown to enhance cardiometabolic health by promoting weight loss, reducing blood pressure, and improving lipid levels [34,35]. Another recently published study highlighted the cardioprotective potential of BHB in various clinical scenarios, including its role in improving cardiac energetics and reducing adverse ventricular remodeling [36].

Moreover, as we have previously mentioned, the combination of sarcopenia and obesity appears to have multiple negative metabolic effects (i.e., especially in older adults); thus, the ketogenic diet could represent a possible synergic intervention to decrease VAT and reduce CVD risk without reducing muscle mass and function [37].

**Figure 2:**
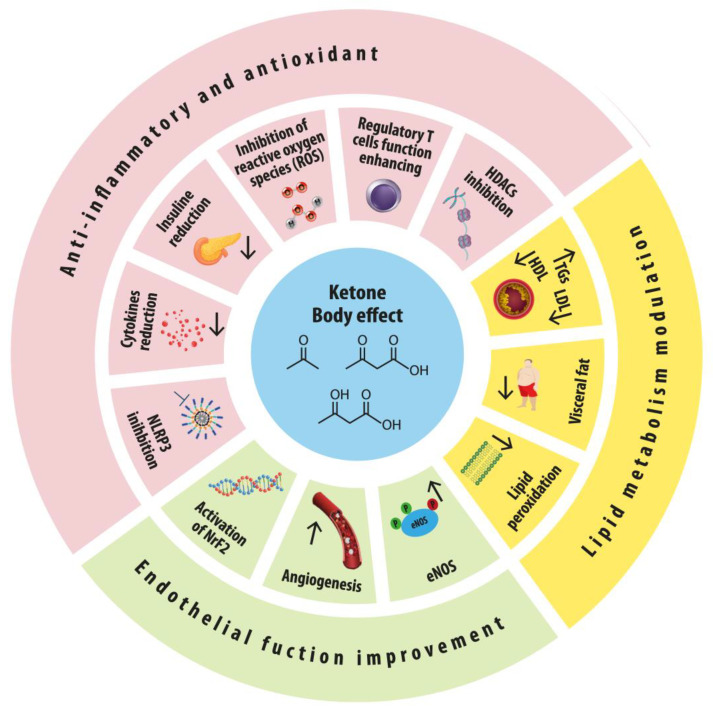
Mechanisms of cardioprotection of nutritional ketosis. The ketone bodies acetoacetate, D-β-hydroxybutyrate, and L-β-hydroxybutyrate, depicted in the center, have pleiotropic effects that act through different mechanisms. Ketone bodies can modulate inflammatory and oxidant states, lipid metabolism, and endothelial function. A subset or combination of these mechanisms underlies the beneficial effects of ketone administration in the setting of cardiometabolic health.

Furthermore, clinical trials have demonstrated that elevated ketone body levels, whether through diet or pharmacological means, are associated with improved cardiovascular outcomes in patients with heart failure [38]. The 2021 review by Puchalska and Crawford also underscores the diverse metabolic and signaling roles of ketone bodies in health and disease, highlighting their potential in therapeutic applications [39]. More recently, the review by Matsuura et al. has provided a comprehensive overview of the metabolic principles and therapeutic implications of ketone bodies in cardiac health. It discusses how ketone bodies serve as a critical cardiac fuel and their diverse roles in regulating cellular processes such as metabolism, inflammation, and cellular crosstalk. Below, we delve deeper into the underlying mechanisms by which ketone bodies, particularly BHB, confer cardiovascular benefits (Figure 2).

### 3.3. Methods to Achieve Nutritional Ketosis

#### 3.3.1. Ketogenic Diet

A ketogenic diet is a high-fat, low-carbohydrate dietary regimen that induces ketosis by mimicking the metabolic state of fasting. Clinical studies have shown significant improvements in weight loss, glycemic control, and lipid profiles among patients following a ketogenic diet [32]. The diet’s ability to significantly lower blood glucose levels and improve insulin sensitivity is particularly beneficial for cardiovascular health [33].

#### 3.3.2. Modified Fasting and Intermittent Fasting

Intermittent fasting (IF) and modified fasting regimens, such as the 5:2 diet, induce ketosis by extending periods of low insulin levels and promoting fatty acid mobilization. These methods improve cardiovascular risk markers, including body weight, blood pressure, and lipid levels [34]. IF has also been linked to improved heart rate variability and reduced markers of oxidative stress [35].

#### 3.3.3. MCT Oil Consumption

Medium-chain triglycerides (MCTs) are rapidly metabolized into ketone bodies, providing a quick source of BHB. Supplementation with MCT oil increases ketone levels and improves metabolic parameters associated with cardiovascular health [40]. Studies have shown that MCT oil can enhance mitochondrial biogenesis and reduce inflammation, further supporting cardiovascular health [41].

#### 3.3.4. Therapeutic Ketosis

Nutritional ketosis typically results in blood ketone levels ranging from 0.5 to 3.0 mmol/L, achieved through dietary modifications such as ketogenic diets or intermittent fasting. This level of ketosis is sufficient to confer various metabolic and cardiovascular benefits without the risk of ketoacidosis [42]. Therapeutic ketosis, on the other hand, is used in clinical settings to manage certain medical conditions such as epilepsy and certain neurodegenerative diseases. Therapeutic ketosis involves higher blood ketone levels, typically between 3.0 to 7.0 mmol/L, achieved through stricter dietary protocols or exogenous ketone supplementation [43]. This higher level of ketosis is carefully monitored to avoid potential adverse effects and is administered under medical supervision. Understanding the distinction between these two levels of ketosis is crucial for tailoring interventions to individual patient needs and optimizing therapeutic outcomes.

## 4. Mechanisms of Cardioprotection of Nutritional Ketosis

### 4.1. Anti-Inflammatory and Antioxidant Effects

Chronic inflammation is a key contributor to atherosclerosis and other cardiovascular conditions. BHB has been found to inhibit the NLRP3 inflammasome, a crucial regulator of inflammatory responses, reducing pro-inflammatory cytokine levels and potentially mitigating inflammation-driven cardiovascular damage [44]. Thus, this inhibition of the NLRP3 inflammasome has been shown to reduce myocardial inflammation and improve cardiac function in models of heart failure [39]. Studies demonstrate that BHB can decrease the levels of IL-1β and IL-18, which are central to the inflammatory process [45]. Additionally, ketone bodies modulate immune responses by enhancing the function of regulatory T cells (Tregs) and influencing the activity of macrophages and T cells. These effects contribute to reduced inflammation and improved immune homeostasis, which are crucial for reducing cardiovascular risk [31]. Importantly, these effects distinguish the ketogenic diet from other dietary approaches that may not produce this ketone-mediated effect.

Moreover, by enhancing mitochondrial biogenesis and efficiency, the KD contributes to reduced oxidative stress, which is a major driver of inflammation, playing a significant role in the pathogenesis of cardiovascular diseases.

BHB increases the production of endogenous antioxidants, such as glutathione, and reduces oxidative stress markers, thereby protecting vascular cells from oxidative damage [46]. Furthermore, BHB has been found to inhibit reactive oxygen species (ROS) production in mitochondria, enhancing cellular resilience to oxidative damage and further limiting oxidative damage and inflammation in high-risk tissues such as the heart and the liver [41]. The antioxidative properties of BHB are crucial in preventing the oxidative modification of LDL cholesterol, which is a key step in the development of atherosclerosis [39]. Moreover, BHB inhibits class I histone deacetylases (HDACs), leading to the increased expression of genes involved in oxidative stress resistance, such as those encoding for antioxidant enzymes like superoxide dismutase (SOD) and catalase. This epigenetic regulation contributes to a reduction in oxidative stress, protecting the cardiovascular system from oxidative damage [31].

The ketogenic diet significantly limits carbohydrate intake, thus reducing postprandial glucose levels and minimizing insulin spikes. Lower insulin levels reduce the expression of pro-inflammatory cytokines and other markers of inflammation [33]. This is particularly beneficial for patients with diabetes and insulin resistance, as stable blood glucose reduces glycation end-products that can trigger inflammatory responses [47].

Nutritional ketosis facilitates weight loss, which has been associated with reductions in visceral fat known, as discussed above, to be particularly detrimental to cardiovascular health [42,48].

### 4.2. Endothelial Function Improvement

Endothelial dysfunction is a precursor to atherosclerosis and is associated with impaired nitric oxide (NO) production. It has been demonstrated that BHB enhances endothelial function by upregulating the expression of endothelial nitric oxide synthase (eNOS), thus improving NO availability and vascular health [49]. Additionally, BHB has been shown to reduce endothelial cell apoptosis and increase angiogenesis, further supporting vascular integrity [50]. This effect is particularly important in maintaining vascular homeostasis and preventing atherosclerotic plaque formation [31,39]. The ketogenic diet has been demonstrated to activate the transcription factor Nrf2 in endothelial cells, inducing the transcription of cellular-antioxidant-defense target genes [51]. Notably, BHB was found to decrease the secretory phenotype associated with vascular cell senescence in aging mice [52]. Moreover, ketosis increased arterial stiffness in children and young adults treated with the diet [53]. The ketogenic diet is also associated with reductions in both systolic and diastolic blood pressure. These effects are thought to be mediated by improvements in endothelial function and reductions in systemic inflammation [54]. Additionally, the diuretic effect of a ketogenic diet may contribute to lower blood pressure [43].

### 4.3. Lipid Metabolism Modulation

Nutritional ketosis influences lipid profiles by increasing high-density lipoprotein (HDL) cholesterol and decreasing triglycerides. BHB and ketogenic diets are associated with favorable shifts in lipid profiles, which are critical for reducing cardiovascular risk [55]. Moreover, BHB has been shown to downregulate lipogenic enzymes and upregulate fatty acid oxidation pathways, further contributing to improved lipid metabolism [56].

Patients with coronary heart disease (CHD) are considered at very high cardiovascular risk, because CHD has been characterized as a chronic immunoinflammatory, fibroproliferative disease fueled by lipids. Accordingly, the last European Society of Cardiology Guidelines suggest that in CHD, the treatment goal is to lower LDL-C levels to <1.4 mmol/L (<55 mg/dL) and achieve a reduction by at least 50% from baseline. For patients who experience a second vascular event within 2 years while taking maximum-tolerated statin-based therapy, an even lower LDL-C goal of <1.0 mmol/L (40 mg/dL) may be considered [57,58,59]. In this framework, the modulation of lipid metabolism induced by the ketogenic diet leads to a reduction in small, dense LDL particles, which are more atherogenic, and an increase in larger, less atherogenic LDL particles [39,60]. It is hypothesized that the increase in LDL particle size associated with ketogenic diets may reduce the atherogenic potential of LDLs [42]. By improving lipid profiles, nutritional ketosis helps reduce overall cardiovascular risk [31].

The ketogenic diet influences lipid metabolism by shifting the body toward the utilization of fat as its primary energy source. In individuals with insulin resistance, lipid accumulation can activate inflammatory pathways [61]. The ketogenic diet promotes lipolysis and reduces ectopic lipid deposition, thereby decreasing lipotoxicity and the associated inflammatory response, which is particularly advantageous for those with CVD. Furthermore, the anti-inflammatory properties and ability of BHB to inhibit lipid peroxidation may slow the progression of plaque formation in arteries, presenting a protective effect against coronary artery disease [56].

## 5. Limitations and Conclusions

The current evidence suggests that ketogenic diets and other methods to achieve nutritional ketosis may offer significant cardioprotective benefits by improving various cardiovascular risk factors.

For patients with cardiovascular disease and diabetes, ketosis presents a dietary strategy that directly addresses inflammatory pathways central to these conditions. By stabilizing blood glucose, reducing insulin secretion, and lowering pro-inflammatory cytokines, the ketogenic diet offers benefits beyond those typically seen with standard hypocaloric diets.

Indeed, unlike calorie-restricted diets, which primarily exert anti-inflammatory effects through weight loss, the ketogenic diet directly targets inflammatory pathways via ketone body production, insulin suppression, and mitochondrial optimization. These specific mechanisms suggest that the ketogenic diet may offer enhanced protection against the progression of inflammation-related metabolic and cardiovascular diseases. Consequently, further research and clinical application of the ketogenic diet for high-risk patients could lead to improved outcomes, particularly in populations with heightened inflammatory profiles.

Nonetheless, its application must be carefully considered due to a range of contraindications and limitations based on patient health profiles. General limitations include potential nutrient deficiencies, particularly in vitamins B and C, magnesium, and fiber, alongside the “keto flu”, which is a collection of symptoms such as fatigue, headache, and nausea occurring during adaptation to ketosis. Moreover, long-term adherence to the ketogenic diet can be challenging due to its restrictive nature, leading to potential weight regain. Indeed, prolonged adherence to the ketogenic diet is generally discouraged in clinical practice due to its potential adverse effects, including micronutrient deficiencies, renal dysfunction, and dyslipidemia, which may compromise its long-term safety.

Therefore, while the ketogenic diet may present notable benefits for particular patient groups (such as for patients with CVD without elevated cholesterol levels), its implementation must be individualized, closely monitored, and accompanied by professional guidance to ensure both efficacy and safety.
